# RF Energy Harvesting–Aided IoT Network: System Design and Prototype Implementation

**DOI:** 10.3390/mi17010137

**Published:** 2026-01-22

**Authors:** Yang Wang, Hangyi Chen

**Affiliations:** 1 Yangtze Delta Region Institute (Huzhou), University of Electronic Science and Technology of China, Huzhou 313001, China; yang.wang@csj.uestc.edu.cn; 2 Zhejiang Huzhou Kaixin Digital Technology Co., Ltd., Hangzhou 310020, China

**Keywords:** Internet of Things (IoT), energy supply, wireless information transfer (WIT), wireless power transfer (WPT), energy harvesting

## Abstract

As more and more Internet of Things (IoT) devices are widely deployed, the issue of energy supply for these devices is becoming increasingly prominent. Considering not only the wireless information transfer (WIT) function of traditional IoT networks but also the characteristics of wireless power transfer (WPT), an RF energy harvesting–aided IoT network is proposed in this paper. In the new IoT network, a WPT transmitter and a WPT receiver are, respectively, introduced to the new gateway and the new end-device. A WPT transmitter is mainly composed of an antenna selection circuit, a power amplifier, and a directional antenna. A WPT receiver consists of a directional antenna, a matching network, a rectification circuit, and an energy management circuit. In order to coordinate WPT and WIT in an orderly manner and minimize WPT’s interference on WIT, a time-division scheme is adopted. The proposed new IoT network aims to offer a new IoT scheme combining both WIT and WPT technologies. In addition, IoT devices can obtain a new energy supply through RF energy harvesting. Both the effectiveness and efficiency of the proposed RF energy harvesting–aided IoT network have been validated through experimentation.

## 1. Introduction

The Internet of Things is currently developing rapidly, with a large number of IoT devices being put into use. For battery powered IoT devices, it is crucial to address their energy supply issues, which can greatly reduce the maintenance cost of replacing batteries for these devices. How to achieve a new energy supply solution for IoT devices and integrate it perfectly with traditional IoT network has become a very popular research field. This article aims to organically combine RF energy harvesting technology with traditional IoT network, proposing a new IoT architecture to solve the energy supply problem of IoT devices.

## 2. Related Work

The Internet of Things has always been very popular, with a large number of researchers conducting research and development work from different perspectives, constantly driving the development of this field [1,2,3]. A comprehensive review of emerging and enabling technologies related to the 5G system that enables IoT is provided in [1]. Many new wireless architectures and smart services attract much attention. How to reduce devices’ energy consumption is a very important research direction in the current Internet of Things [4]. This paper [4] discusses the design and testing of LoRa communication-based Internet-of-Things devices to track relatively small and inexpensive non-powered assets and presents an innovative method to minimize LoRa module energy by monitoring for localized movement or acceleration as well as changes in the received signal strength from the base station. By taking into account not only the node specifics but also the benefits of mobile edge computing, an integrated strategy for energy saving in Internet of Things devices is proposed in this article [5]. The proposed integrated solution aims to decrease the energy consumption of IoT devices as much as possible and thus prolong their battery lives.

Electromagnetic waves can not only carry information but also carry energy. More and more researchers are exploring the energy properties of radio frequency signals and utilizing them [6,7,8,9,10,11,12,13]. The authors [6] propose performing harvesting operations at typical ambient radio frequency power levels found within urban environments. The proposed harvester includes an antenna, an impedance-matching network, and a rectifier. Numerical and experimental data are reported and discussed to show the effectiveness of RF energy harvesting. In order to achieve stable power output and constant transfer efficiency under different coupling capacitances, the circuit model of the capacitive wireless power transfer (CPT) system with a double-sided LC compensation network is established in [10]. The potential advantages of applying a reconfigurable intelligent surface (RIS) in wireless power transfer systems are investigated, and an efficient RIS-aided WPT scheme is designed in [11]. This article [12] reviews the application of machine learning (ML) techniques in wireless power transfer systems, focusing on their role in optimizing system performance, enhancing safety, and improving efficiency. This paper [13] studies wireless electric vehicle charging (WEVC) technology and the mentioned thermal risks in wireless EV chargers, considering spatial misalignment, providing valuable insights for WPT based on RF signals.

In addition to studying data transmission and energy transmission, respectively, many researchers are now attempting to combine the two. Researchers generally refer to this technology as Simultaneous Wireless Information and Power Transfer (SWIPT) [14,15,16,17,18,19], while the new network architecture that applies this technology is commonly referred to as the Data and Energy Integrated Network (DEIN) [20]. A novel co-designing strategy is presented for an integrated rectifying metantenna, which combines rectification, absorption, and electromagnetic signal reception functionalities for simultaneous wireless information and power transfer in [17]. The proposed rectifying metantenna boasts a range of merits, including high integration and multifunctionality. How to coordinate WIT and WPT in a SWIPT system is also widely studied. This article [21] proposes maximizing the sum energy efficiency (EE) of all device-to-device (D2D) links in a D2D underlaid cellular network by optimizing the resource and power allocation based on a nonlinear energy harvesting (EH) model. This paper [22] proposes a novel system operation sequence for a sensor-cloud system in which sinks opportunistically provide SWIPT to sensor nodes during the downlink phase and collect data transmitted from sensor nodes in the uplink phase, so that energy efficiency in the proposed SWIPT system is clearly optimized. Some prototype work has also been carried out [23,24]. The system designed in this article [23] has some redundancy. For example, the Zigbee router can actually be omitted, and the Zigbee coordinator is sufficient to complete the work of WIT and WPT. The system designed in article [24] is relatively complex. Energy transmitters generally require a high-power energy supply, making it difficult to find proper application scenarios when mounted on a UAV, which has very limited energy storage. Our article provides a more reasonable design scheme below.

The remainder of this paper is organized as follows. System design is illustrated in Section 3 from theory to practice, including the principle of wireless energy transfer, the overall architecture of the proposed new IoT network, the design of the proposed gateway, and the design of the proposed end-device. Prototype implementation of the proposed gateway and the proposed end-device is, respectively, presented in Section 4. Performance evaluation is carried out from different perspectives in Section 5. Finally, the paper comes to a conclusion in Section 6.

## 3. System Design

### 3.1. Wireless Power Transfer

**Figure 1:**
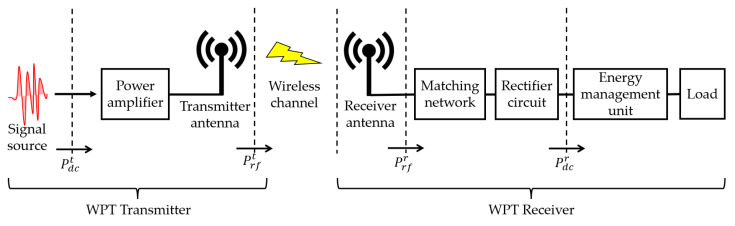
The transmission chain of a WPT system.

The transmission chain of a WPT system is depicted in Figure 1.

A WPT system mainly consists of two parts: a wireless RF energy transmitter and a wireless RF energy receiver. From the signal source on the transmitter side to the final energy that can be used to power the load on the receiver, the entire wireless RF energy transmission chain is composed of multiple important parts:

• Power amplifier: This component is used to amplify the power of the RF signal source for transmission. Power amplifiers have a certain input power range, and it is very important to select and use them reasonably. Here, the power of the signal source is denoted as $P_{dc}^{t}$. The power of the signal emitted through the transmitter antenna is denoted as $P_{rf}^{t}$
• Wireless channel: RF signals will inevitably experience attenuation when propagating in the air, which is mainly caused by path loss and multipath fading. Generally, directional beams and other methods can be used to reduce this impact.
• Matching network: The RF signal received by the receiver may cause an impedance mismatch due to frequency offset. The quality of the impedance matching circuit design has a significant impact on the efficiency of RF energy transfer. Here, the signal power input to the matching network is denoted as $P_{rf}^{t}$
• Rectifier circuit: The RF signal received by the receiver is still an AC signal after passing through the matching network. The rectification circuit is designed to rectify the AC signal into a DC signal that the receiver can utilize. Here, the signal power output by the rectification circuit is denoted as $P_{dc}^{r}$
• Energy management unit: The DC signal power output by the rectifier circuit is generally too low to directly drive the load, so it needs to be aligned and stabilized by the energy management circuit before it can power the electronic load.

Therefore, the energy transfer efficiency of a wireless RF energy transfer system can be denoted as follows:

(1) $$\rho =\frac{P_{dc}^{r}}{P_{dc}^{t}}=\frac{P_{rf}^{t}}{P_{dc}^{t}}\frac{P_{rf}^{r}}{P_{rf}^{t}}\frac{P_{dc}^{r}}{P_{rf}^{r}}$$

From the above equation, it can be seen that in order to fundamentally improve the transmission efficiency of wireless RF energy, every part in the complete transmission chain needs to be carefully designed and optimized.

### 3.2. Overall Architecture

**Figure 2:**
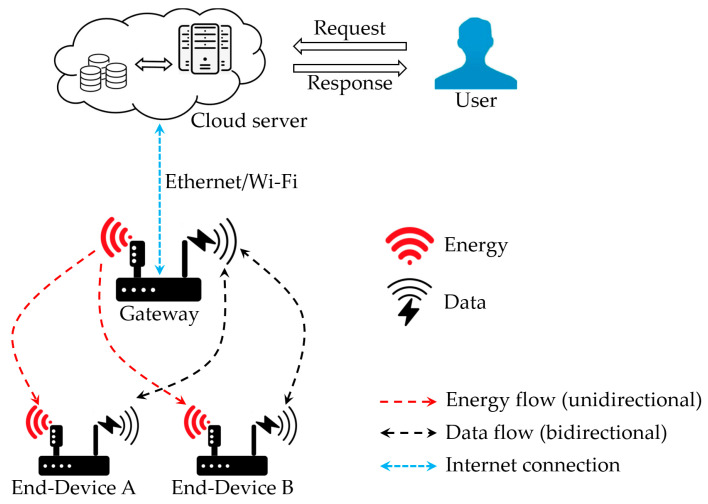
System architecture of an RF energy harvesting–aided IoT network.

The system architecture of the RF energy harvesting–aided IoT network designed in this paper is illustrated in Figure 2. Such an IoT network consists of multiple end-devices, a gateway, and a cloud server. The gateway proposed in this article integrates the general functions of traditional IoT gateways and the wireless energy transmission function of energy transmitters. The gateway creates and maintains the network, and then the end-devices can join the network after obtaining authentication and permission from the gateway. The gateway connects to the Internet through Ethernet or Wi-Fi, so that cloud services can be obtained. Meanwhile, users can remotely control and monitor all end-devices in the IoT network. Both the gateway and end-devices are designed with two antennas: an omnidirectional antenna for wireless data transmission and a directional antenna for wireless energy transmission. For the RF energy harvesting–aided IoT network system shown in Figure 2, the data transmission process and energy transmission process are carried out in a time-sharing manner. In wireless energy transmission mode, the directional antenna used for RF energy transmission on the gateway is enabled, while the omnidirectional antenna used for data communication is disabled. When the RF energy reaches the end-device side, the directional antenna connected to the RF signal rectification and matching circuit on the end-device will absorb energy from the RF signal. Then it will be converted into DC electrical energy and stored for further use. Similarly, in wireless data transmission mode, the omnidirectional antennas on both the gateway and end-devices are enabled, while the directional antennas are disabled. End-devices in such IoT networks are usually wireless battery-less devices, so they must always operate in ultra-low power mode.

### 3.3. Design of Gateway

**Figure 3:**
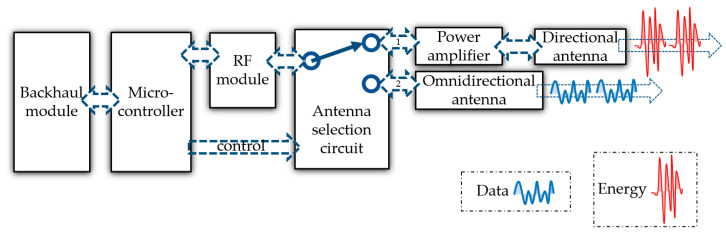
Schematic diagram of the proposed gateway.

Referring to the description of the transmitter part in the transmission chain of a WPT system mentioned earlier, the design principle of the proposed gateway in this article is depicted in Figure 3.

The proposed gateway in this article is mainly composed of several parts below.

1. Backhaul module: It is designed to connect the system to the Internet and obtain cloud services.
2. Microcontroller: It comprehensively controls all the other modules, such as controlling the RF module to generate WIT data packets or WPT energy packets.
3. RF module: It is connected to the microcontroller to generate data packets and energy packets as required.
4. Antenna selection circuit: It can guide the RF signal to different antennas for WPT or WIT, controlled by the microcontroller. For energy packets dedicated to WPT, the RF signal needs to be switched to path 1. For data packets dedicated to WIT, the RF signal needs to be switched to path 2.
5. Power amplifier: It is designed to amplify the RF signal generated by the RF module in order to carry more energy for WPT.
6. Directional antenna: It is connected to the RF link of WPT and used for directional energy transmission to achieve higher energy transmission efficiency.
7. Omnidirectional antenna: It is connected to the RF link of WIT and used for wireless data transmission, which is no different from conventional wireless communication antennas.

### 3.4. Design of End-Device

**Figure 4:**
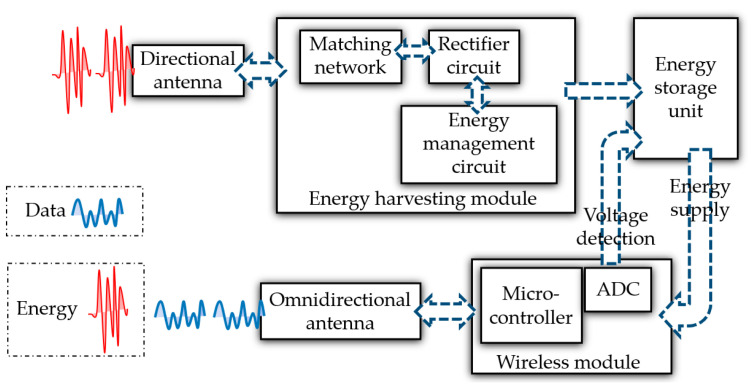
Schematic diagram of the proposed end-device.

Referring to the description of the receiver part in the transmission chain of a WPT system mentioned earlier, the design principle of the proposed end-device in this article is depicted in Figure 4.

The proposed end-device in this article is mainly composed of several parts below.

1. Directional antenna: It matches the WPT link of the proposed gateway. It is helpful to achieve higher energy transmission efficiency by using a directional antenna for energy reception.
2. Energy harvesting module: It is designed to convert RF energy into DC energy and store it. This module consists of a matching network, a rectification circuit, and an energy management circuit. The obtained DC energy can be used to charge energy storage devices such as lithium batteries and supercapacitors, and to finally power loads such as wireless modules.
3. Energy storage unit: it is commonly lithium batteries or supercapacitors.
4. Wireless module: it generally has much lower computing and task requirements than the gateways, so there is no need to add an additional microcontroller like gateways. It is used to simultaneously complete wireless data transmission and reception, as well as processing and controlling tasks. In addition, the wireless module also needs to regularly monitor the energy level of the energy storage unit. If the energy level is below the warning threshold, the wireless module will initiate a WPT request to the proposed gateway to obtain a new energy supply.
5. Omnidirectional antenna: It is similar to the antenna used for data transmission on the proposed gateways.

## 4. Prototype Implementation

### 4.1. Implementation of Gateway

**Figure 5:**
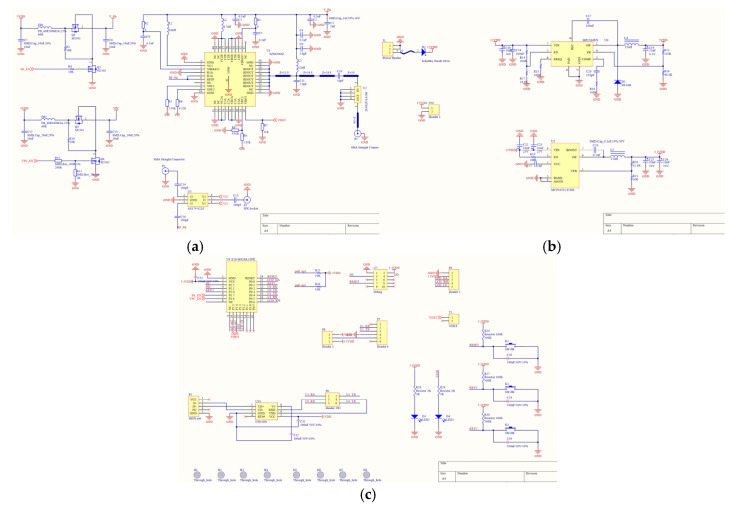
Schematic design of the proposed gateway. (**a**) Part 1; (**b**) part 2; (**c**) part 3.

**Figure 6:**
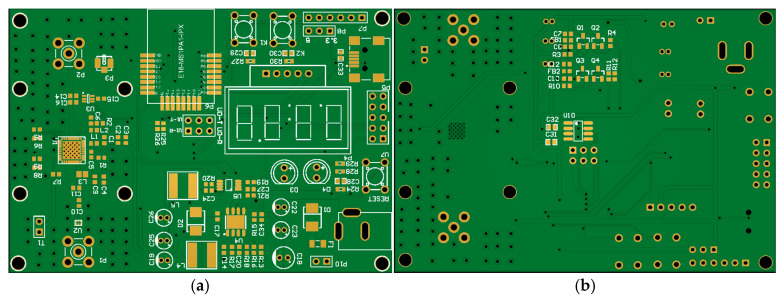
PCB design of the proposed gateway. (**a**) front side; (**b**) back side.

**Figure 7:**
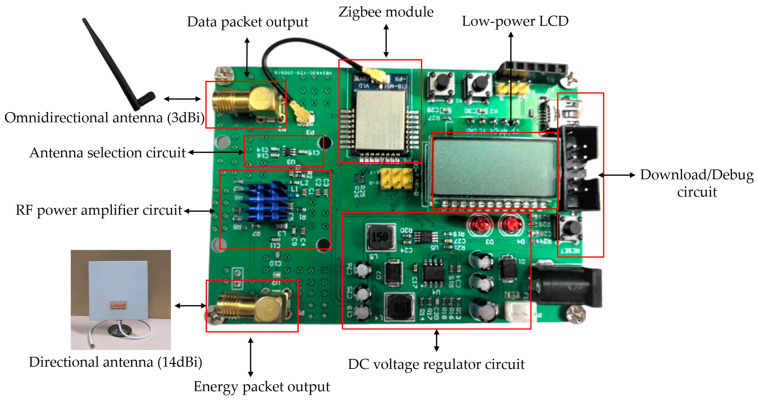
Prototype of the proposed gateway.

According to the design principle in Figure 3, a physical prototype of the proposed gateway is implemented. In order to focus on the collaborative effect of WIT and WPT, the backhaul module is omitted in the actual prototype. The schematic design, PCB design, and prototype are, respectively, shown in Figure 5, Figure 6 and Figure 7.

A brief introduction to the main parts of the prototype is listed below.

1. Zigbee module: the core chip of this module is CC2530 from Texas Instruments (Dallas, TX, USA), which has an embedded microcontroller inside. The software protocol stack and programming methods of this chip are dedicatedly investigated, which have enough processing capabilities to effectively control the whole device. Thus, functions of the microcontroller in the earlier design principle are also incorporated into the CC2530 chip in actual production. By programming the Zigbee module, centralized control and coordination of all other end-devices have been fully achieved, along with response to their WIT and WPT requests.
2. Antenna selection circuit: It is implemented with the Zigbee module controlling AS17992LFfrom Skyworks Solutions (Irvine, CA, USA), which can switch the RF signal generated by the wireless module to a low-gain and low-power data transmission chain, or to a high-power and high-gain energy transmission chain at will. Therefore, WPT and WIT are implemented in a time-division manner.
3. Energy link: it consists of an RF power amplifier and a high-gain directional antenna. The RF signal originally generated by the wireless module does not have enough power to activate the rectifier of the receiver. With the help of the RF power amplifier chip SZM-2066Zfrom Qorvo (Greensboro, NC, USA), RF signals from 2.4 GHz to 2.7 GHz can be amplified to enable effective energy transmission.
4. Data link: an omnidirectional antenna is used for transmitting and receiving low-power WIT signals.
5. DC voltage regulator circuit: When the proposed gateway performs wireless energy transmission, the RF power amplifier chip SZM-2066Z requires high electric power. The DC conversion chip MP1584from Monolithic Power Systems (Kirkland, WA, USA) is used to generate constant voltages of 5 V and 3.3 V, and can provide a maximum rated current of 3 A, ensuring a stable DC energy supply for the whole system.

### 4.2. Implementation of End-Device

**Figure 8:**
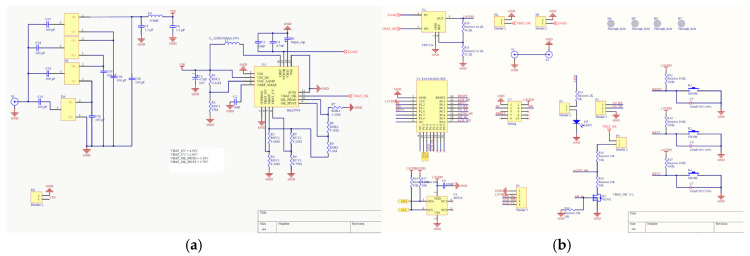
Schematic design of the proposed end-device: (**a**) part 1; (**b**) part 2.

**Figure 9:**
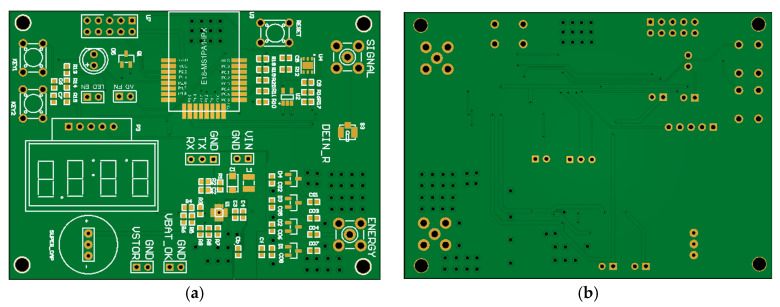
PCB design of the proposed end-device: (**a**) front side; (**b**) back side.

**Figure 10:**
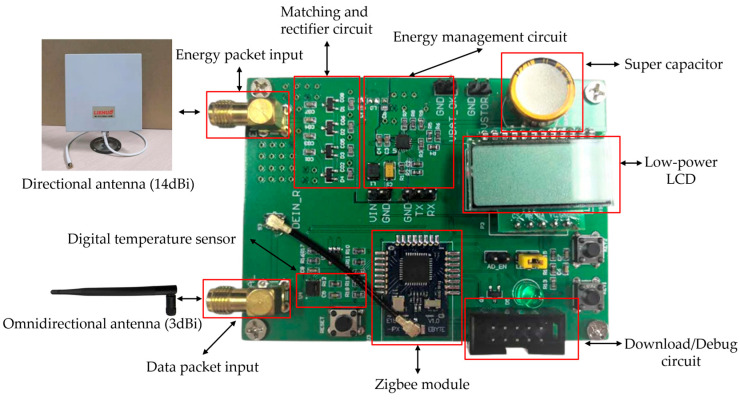
Prototype of the proposed end-device.

According to the design principle in Figure 4, a physical prototype of the proposed end-device is implemented. The schematic design, PCB design, and prototype are, respectively, shown in Figure 8, Figure 9 and Figure 10.

Details of the main parts of the prototype above are listed below.

1. Zigbee module: similar to the actual prototype of the proposed gateway, the CC2530 chip is used in the prototype of the proposed end-device, of which the wireless data transmission and data processing functions are fully utilized. The complete Zigbee protocol is running on this module, and it can communicate with the gateway according to the standard Zigbee protocol. The module will also transmit the monitoring results of the local power level to the gateway according to the Zigbee protocol.
2. Digital temperature sensor: its specific model is SHT20from Sensirion (Stäfa, Switzerland) with an I2C interface. It is a high-precision but low-power digital temperature and humidity sensor that can effectively monitor environmental temperature.
3. Antenna: the omnidirectional antenna and directional antenna of this prototype share the same model as the proposed gateway, which can achieve reasonable data and energy transmission effects.
4. RF energy harvesting module: it consists of a matching and rectifier circuit, an energy management circuit, and a supercapacitor. RF energy can be converted into direct current and stored in supercapacitors. With the implementation of an energy management circuit based on the chip BQ25504 from Texas Instruments (Dallas, TX, USA), it can provide a stable energy supply for all loads on the prototype.

## 5. Experimentation and Analysis

This section will introduce the construction of the experimental system with the prototypes mentioned earlier and carry out experimental analysis from different perspectives.

### 5.1. Experimental System

**Figure 11:**
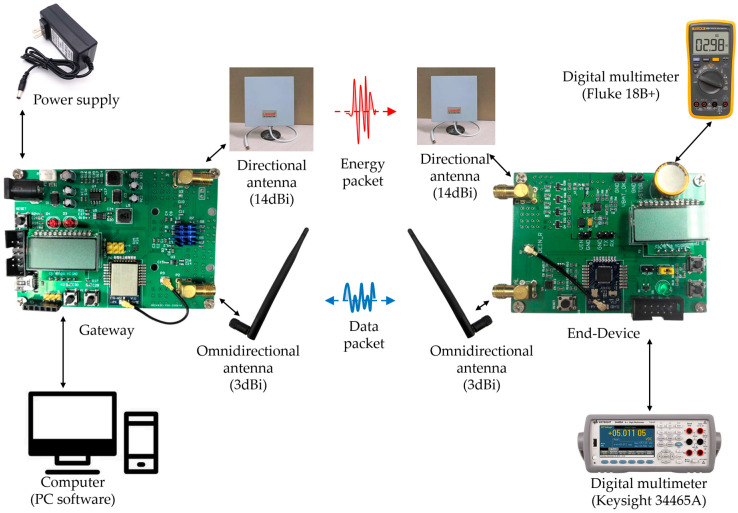
The experimental system.

The experimental system built is shown in Figure 11, mainly composed of a gateway and an end-device.

A 14 dBi high-gain planar directional antenna and a 3 dBi low-gain rubber rod omnidirectional antenna are connected to the gateway for WPT and WIT, respectively. The two directional antennas must be strictly aligned to minimize the loss of RF energy as much as possible. The power adapter converts 220 V AC mains power to 12 V DC power to provide stable and reliable power to the gateway. In addition, the gateway is connected to the computer through the debugging port reserved on the circuit board. The software running on the computer can not only monitor the system’s running log, but also control the gateway. On the end-device side, the antenna connection is consistent with that of the gateway. However, in this experiment, the end-device is a passive device without a battery or wired power supply, so it is not connected to any power source. In addition, the energy collection on the end-device is monitored using digital multimeter 18B+ from Fluke (Everett, WA, USA) and digital multimeter 34465A from Keysight (Santa Rosa, CA, USA), respectively, for real-time numerical display and data recording.

The key RF parameters used in this experimental system are shown in Table 1. It needs to be specifically noted that, in order to meet the demand for data and energy coordinated transmission, the system uses the time-division method to carry out WPT and WIT. At the same time, this method can also minimize the interference of WPT on WIT. When the gateway enters the WPT mode, it generates and sends RF energy packets within one working cycle. Then it immediately switches to WIT mode for data services. Once a whole working cycle ends, the gateway will start a new round of RF energy packet transmission.

The gateway designed and implemented in this article integrates the general functions of traditional IoT gateways and the wireless RF energy transmission function. After powering on, the gateway will automatically create a Zigbee network through the onboard Zigbee module. As the creator of this wireless network, the gateway also needs to maintain it, for example, by managing the authentication and joining of new end-devices. When energy transmission is required, the proposed gateway will automatically generate wireless RF energy packets, which are wirelessly output through connected power amplifiers and directional antennas to offer power supply to the proposed end-devices. From the perspective of data transmission, this end-device is essentially a low-power Zigbee device. When the device obtains a certain initial energy through wireless means, it can start working. A high-precision, low-power digital temperature sensor, SHT20, is mounted on the end-device. Once the end-device successfully pairs with the gateway, it will start periodically collecting temperature data from the environment and uploading it to the gateway. A portable digital multimeter, 18B+ from Fluke, and a multifunctional digital multimeter, 34465A from Keysight, are used in this experimental part, mainly for monitoring and recording the real-time voltage level of the super capacitor on the proposed end-device.

### 5.2. Effectiveness Verification

**Figure 12:**
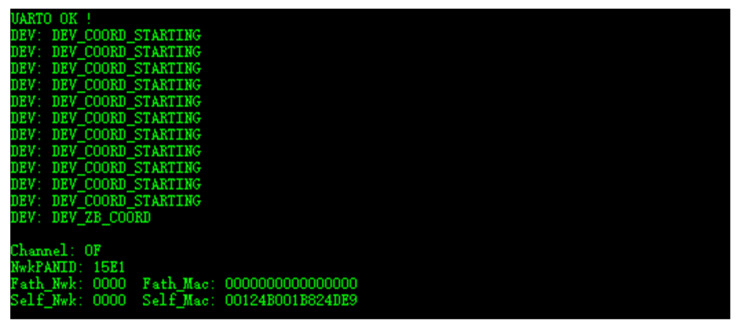
Log of the gateway startup.

After the experimental system based on the gateway and end-device is built, the gateway is powered on and connected to the debugging software on the computer through the reserved debugging interface onboard. The startup information on the computer screen is shown in Figure 12.

From Figure 12, it can be seen that the serial port of the debugging interface is initialized first, and then a Zigbee network is automatically created. This Zigbee network is established on channel 15 (Zigbee channel 15 corresponds to a frequency of 2425 MHz) and has a network identifier of 0x15E1.

**Figure 13:**
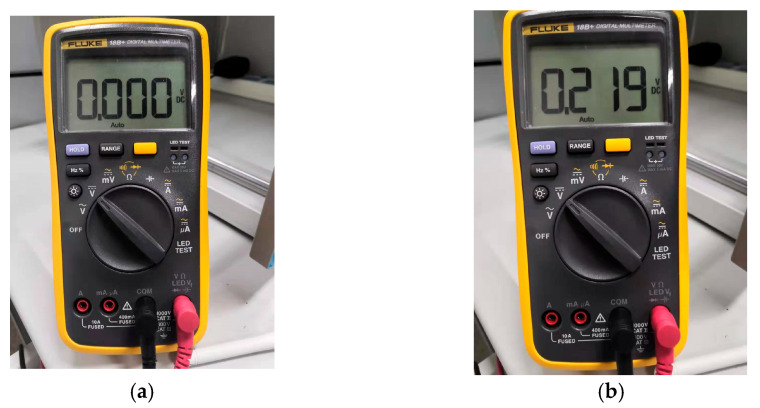
Voltage of the super capacitor on the end-device: (**a**) when WPT starts; (**b**) 30 s later.

Due to the fact that the end-device is a passive device with no initial energy, the first energy transmission must be initiated by the user controlling the gateway to initiate downlink RF energy transmission. The distance between the gateway and the end-device is 0.35 m, and the RF transmission power of the gateway is 1.5 watts. The wireless charging of the end-device is started with debugging software on the computer controlling the gateway. The Fluke digital multimeter is connected to the two ends of the super capacitor on the end-device, and the changes in measured voltage values are shown in Figure 13.

The target voltage for wireless charging is 3.1 V, which is achieved through hardware programming of the energy management circuit on the end-device. This value is set because it is the voltage threshold required for the CC2530 chip to operate. The preset value is slightly lower than the standard 3.3 V to enable the system to operate at a lower voltage and consume less energy.

**Figure 14:**
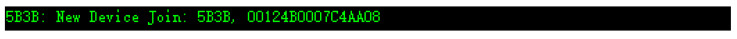
Log of the end-device joining the network.

As shown in Figure 13, during the wireless charging process, the voltage of the super capacitor on the end-device gradually increases, indicating that the end-device has successfully received the wireless RF energy packets and converted them into DC energy. When the voltage of the super capacitor rises to the preset threshold of 3.1 volts, the end-device starts working. Due to the default setting of the Zigbee network being always open after creation, the end-device successfully joins the network. From the software interface on the computer, the corresponding log of the new device’s network connection can be seen, as shown in Figure 14. From Figure 14, it can be seen that the network short address of the newly connected end-device is 0x5B3B, and the MAC address is 0x00124B0007C4AA08.

**Figure 15:**
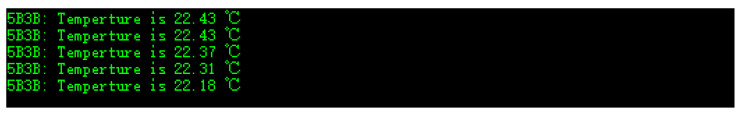
Log of environmental temperature data.

After joining the network, the end-device starts to periodically collect environmental temperature data and upload it to the gateway. From the software interface on the gateway side, it can be seen that the upstream sensor data received by the gateway comes from the end-device with device address of 0x5B3B, which is a newly connected end-device, as shown in Figure 15. The measured temperature of the environment is basically around 22 °C. Accordingly, the effectiveness of the experimental system has been verified through the downlink RF energy transmission of the gateway, the energy reception of the end-device, and the final uplink data transmission of the end-device, which confirms the effectiveness of the RF energy harvesting–aided IoT network.

### 5.3. Performance Evaluation

**Figure 16:**
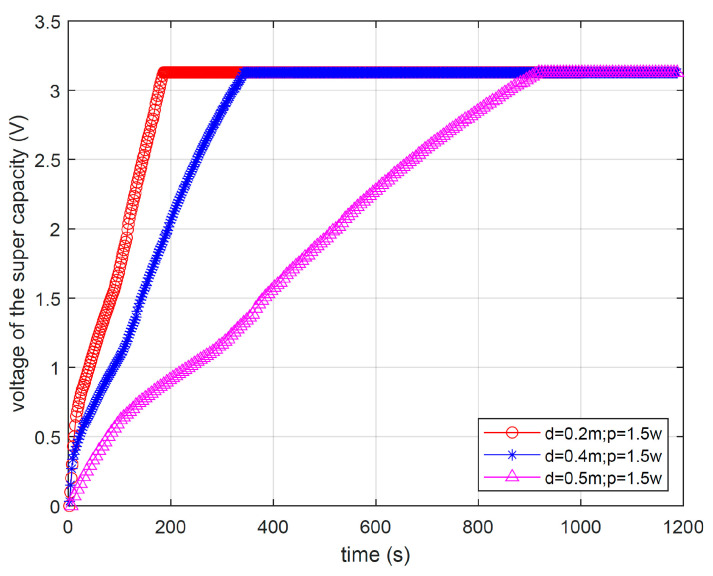
Voltage changes with different distances.

**Figure 17:**
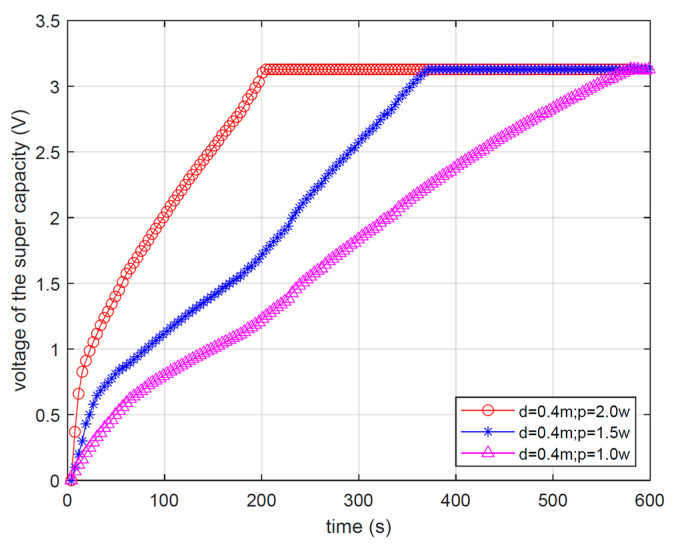
Voltage changes with different transmission powers.

The RF transmission power of the gateway can be adjusted arbitrarily within a certain range. Both Figure 16 and Figure 17 depict the voltage variation in super capacitors on the end-device over time. The two figures, respectively, demonstrate the impact of the distance between the gateway and the end-device, as well as the RF energy transmission power of the gateway, on the wireless RF energy transmission effect.

Figure 16 shows the impact of different distances on RF energy transmission when the RF energy transmission power is fixed at 1.5 w. From Figure 16, it can be seen that increasing the transmission distance will require more time for the end-device to reach the preset target voltage. For example, when the transmission distance is 0.2 m, the super capacitor on the end-device only takes 190 s to reach the target voltage of 3.1 V. But when the transmission distance increases to 0.5 m, it takes about 900 s to reach the target voltage. This is because longer transmission distances result in higher path losses for RF signals during transmission, directly leading to more energy loss. Therefore, the charging time will significantly increase with the increase in distance.

Figure 17 shows the impact of different RF energy transmission powers on RF energy transmission when the distance between the gateway and the end-device is fixed at 0.4 m. From Figure 17, it can be seen that when other conditions remain constant, the higher the RF energy transmission power is, the less time the wireless charging process will take. For instance, when the RF energy transmission power increases from 1.5 W to 2 W, the time for the super capacitor on the end-device to reach the target charging voltage decreases from 360 s to 200 s. This is because the higher the transmission power of RF energy is, the more energy the RF signal reaching the end-device will have.

## 6. Conclusions

This paper first introduces the principle of wireless RF energy transmission and its key components. Then, based on the principle of WPT, detailed design schemes and corresponding prototype implementation details of the gateway and end-device are proposed, including chip selection for key components. Finally, based on the RF energy harvesting–aided IoT network designed in this paper, experiments from different perspectives are designed and carried out. The effectiveness of the proposed new IoT network is verified from the perspective of downlink RF energy transmission and uplink data reports. The performance of RF energy transmission is also analyzed. This paper demonstrates the implementation of a prototype system using readily available and low-cost components, rather than relying on expensive ones. This approach makes technology significantly more accessible for widespread adoption. In the future, the two antennas on the end-device can be merged into one, then the received RF signal can be divided into two parts with a power splitter: one part used for energy harvesting and the other part used for information decoding. In that case, it will be closer to the final state of Simultaneous Wireless Information and Power Transfer.

## Figures and Tables

**Table 1:** RF parameters of key components in the experimental system.

No.	Item	Value
1	Max power of the power amplifier	2 W
2	Frequency of the directional antenna	2.4 GHz
3	Gain of the directional antenna	14 dBi
4	Size of the directional antenna	17 cm × 17 cm × 1.3 cm
5	Frequency of the omnidirectional antenna	2.4 GHz
6	Gain of the omnidirectional antenna	3 dBi
7	Size of the omnidirectional antenna	11 cm × Ø1 cm
8	Distance between the two directional antennas	60 cm
9	Distance between the two omnidirectional antennas	40 cm
10	Time interval between two adjacent	30 ms

## Data Availability

The original contributions presented in the study are included in the article, further inquiries can be directed to the corresponding author.
